# The Tip of the Four *N*-Terminal α-Helices of *Clostridium sordellii* Lethal Toxin Contains the Interaction Site with Membrane Phosphatidylserine Facilitating Small GTPases Glucosylation

**DOI:** 10.3390/toxins8040090

**Published:** 2016-03-25

**Authors:** Carolina Varela Chavez, Georges Michel Haustant, Bruno Baron, Patrick England, Alexandre Chenal, Serge Pauillac, Arnaud Blondel, Michel-Robert Popoff

**Affiliations:** 1 Unité des Bactéries anaérobies et Toxines, Institut Pasteur, 75724 Paris cedex15, France; carolina.varela-chavez@igh.cnrs.fr (C.V.C.); georges.haustant@pasteur.fr (G.M.H.); serge.pauillac@pasteur.fr (S.P.); 2 Plate-Forme de Biophysique Moléculaires, Institut Pasteur, 75724 Paris cedex15, France; bruno.baron@pasteur.fr (B.B.); patrick.england@pasteur.fr (P.E.); 3 Unité de Biochimie des Interactions Macromoléculaires, Institut Pasteur, 75724 Paris cedex15, France; alexandre.chenal@pasteur.fr; 4 Unité de Bioinformatique Structurale, Institut Pasteur, 75724 Paris cedex15, France; ablondel@pasteur.fr

**Keywords:** *Clostridium sordellii*, *Clostridium difficile*, large clostridial glucosylating toxin, *C. sordellii* lethal toxin, phosphatidylserine, Ras

## Abstract

*Clostridium sordellii* lethal toxin (TcsL) is a powerful virulence factor responsible for severe toxic shock in man and animals. TcsL belongs to the large clostridial glucosylating toxin (LCGT) family which inactivates small GTPases by glucosylation with uridine-diphosphate (UDP)-glucose as a cofactor. Notably, TcsL modifies Rac and Ras GTPases, leading to drastic alteration of the actin cytoskeleton and cell viability. TcsL enters cells via receptor-mediated endocytosis and delivers the *N*-terminal glucosylating domain (TcsL-cat) into the cytosol. TcsL-cat was found to preferentially bind to phosphatidylserine (PS)-containing membranes and to increase the glucosylation of Rac anchored to the lipid membrane. We have previously reported that the *N*-terminal four helical bundle structure (1–93 domain) recognizes a broad range of lipids, but that TcsL-cat specifically binds to PS and phosphatidic acid. Here, we show using mutagenesis that the PS binding site is localized on the tip of the four-helix bundle which is rich in positively-charged amino acids. Residues Y14, V15, F17, and R18 on loop 1, between helices 1 and 2, in coordination with R68 from loop 3, between helices 3 and 4, form a pocket which accommodates L-serine. The functional PS-binding site is required for TcsL-cat binding to the plasma membrane and subsequent cytotoxicity. TcsL-cat binding to PS facilitates a high enzymatic activity towards membrane-anchored Ras by about three orders of magnitude as compared to Ras in solution. The PS-binding site is conserved in LCGTs, which likely retain a common mechanism of binding to the membrane for their full activity towards membrane-bound GTPases.

## 1. Introduction

*Clostridium sordellii* is responsible for severe traumatic infections, as well non-traumatic myonecrosis and toxic shock in man and animals. Although *C.* *sordellii* infections sporadically occur in humans, they are rapidly dramatic. Notably, *C.* *sordellii* infection of the genital tract in women results in a fulminant toxic shock syndrome [1,2,3,4]. In addition, *C.* *sordellii* causes rapidly fatal enterotoxemia in livestock. *C.* *sordellii* lethal toxin (TcsL) is the major virulence factor of this pathogen. TcsL belongs to the large clostridial glucosylating toxin (LCGT) family which encompasses *Clostridium difficile* toxin A (TcdA) and toxin B (TcdB), *Clostridium* *novyi* alpha toxin (TcnA), and *Clostridium perfringens* large cytotoxin (TpeL) [5,6,7]. Some *C.* *sordellii* strains produce the hemorrhagic toxin (TcsH) in addition to TcsL [8,9].

LCGTs are large single chain proteins (MW, 250–300 kDa), which are active intracellularly. LCGTs catalyze glucosylation of small GTPases from the Rho and/or Ras family at the conserved Thr35/37, yielding inactive signal molecules [10]. While *C. difficile* and *C. sordellii* toxins use UDP-glucose as a cofactor, TcnA and TpeL prefer UDP-*N*-acetylglucosamine [10,11,12,13,14,15,16]. Each LCGT interacts with distinct sets of GTPases [7,8,17,18,19,20]. Thereby, TcsL mainly targets Rac and Ras proteins and induces actin cytoskeleton depolymerization and cytotoxicity [8,21]. Rac inactivation by LCGTs is the main player in mediating actin cytoskeleton disorganization and, subsequently, intercellular junction alteration via paxillin dephosphorylation and controlling phosphoinositide cell content [8,22,23], whereas TcsL-dependent Ras inactivation induces apoptosis [21,24].

LCGTs contain several structural and functional domains which are involved in the sequential steps of their mode of action. LCGTs *C*-terminal domain contains combined repetitive oligopeptides (CROPS) which are supposed to interact with cell surface receptors. However, *C.* *difficile* toxin B (TcdB) binds to its receptors which have been identified as poliovirus receptor-like 3 [20,25] and chondroitin sulfate proteoglycan 4 [26], via a receptor-binding domain upstream of CROPS [26,27]. Upon binding to their receptor(s), LCGTs enter cells via clathrin-dependent endocytosis. Endosome acidification triggers a conformational change of the translocation domain which inserts into the endosomal membrane and forms small pores resulting in the translocation of the catalytic (cat) *N*-terminal domain into the cytosol. Autocleavage of the cat domain via the cysteine protease site (DHC) is activated by host inositol hexakisphosphate [20,27,28,29]. Therefore, translocation of the catalytic domain into the cytosol and its subsequent subcellular localization allowing the interaction with its substrates are a critical step in the intoxication process. Small GTPases cycle between GTP-bound active and GDP-bound inactive forms. Active and inactive Ras proteins are anchored to the plasma membrane [30], whereas Rho family GTPases cycle between active forms bound to the plasma membrane, and inactive forms which are linked to GDI (guanine nucleotide dissociation inhibitor) in the cytosol. Activation by a guanine nucleotide exchange factor (GEF) leading to GDP-GTP exchange takes place at the cell membrane where the GTPases dissociated from GDI are anchored through a *C*-terminal isoprenyl moiety [5]. Thereby, the interaction of LCGT catalytic domains with the membrane-bound substrates is required for their full activity. Membrane targeting is an important aspect of many proteins that have a specific function at the membrane level or participate in a specific membrane structure [31]. Various bacterial protein toxins, which are active inside cells, notably by interacting with a membrane bound compound, retain a membrane-binding motif. This is the case of toxins, which target small GTPases such as LCGTs and multifunctional-autoprocessing repeats-in-toxins (MARTX), or heterotrimeric GTPases, such as *Pasteurella multocida* toxin (PMT) [32,33,34,35,36]. Indeed, the 1–93 domain of LCGTs, which contains the *N*-terminal four-helix bundle has been shown to have a critical role in the binding of the catalytic domains to negatively-charged phospholipids [33,34,37,38]. Indeed, deletion of the 18 *N*-terminal amino acids abolishes the binding of TcsL-cat to PS-containing liposomes [38].

The overall structure of the cat domains (amino acids 1-543) of TcdB, TcsL, and TcnA is conserved and consists in a β-strain central core (about 235 amino acids) forming an active center pocket with the catalytic site DXD surrounded by numerous α-helices [39,40]. We previously found that TcsL binds to phosphatidylserine (PS) and that the 19 *N*-terminal amino acids of TcsL are required for the interaction with the lipid [38]. This structure has been found to bind to membranes using its positively-charged residues [33,34]. We have reported that TcsL-cat selectively binds to PS and phosphatidic acid (PA), whereas TcsL 1–93 domain interacts with a broad range of lipids [37]. Here, we identify by generating mutations the residues of TcsL 1–93 domain involved in PS binding and localization of the cat domain to the membrane by mutagenesis.

## 2. Results

### 2.1. Hydrophobic Patches on TcsL 1–93 and Mutation Selection

**Figure 1:**
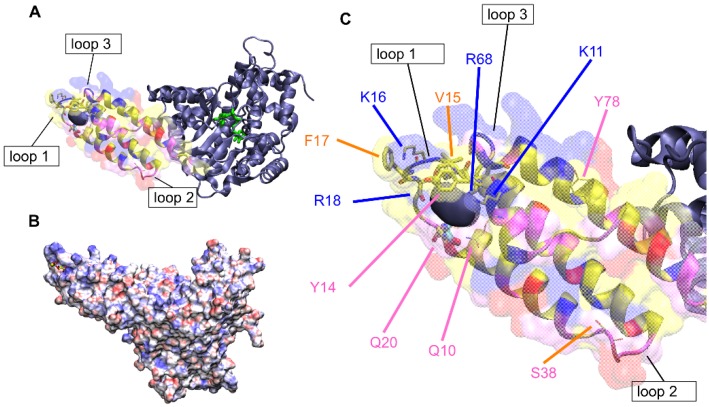
Localization of the hydrophobic patches and mutated positions on the TcsL *N*-terminus. The protein is displayed in ribbons; the UDP-glucose in green sticks. Amino acids of the *N*-terminal domain are colored by type: positively-charged in blue; negatively-charged in red; polar in pink; hydrophobic in yellow or orange. A hollow surface display the solvent-excluded volume for this domain and the solid volume in ice-blue marks a cavity at the surface of the crystal structure (chain A of pdb 2VDK). (**A**); TcsL-cat; (**B**). TcsL-cat, surface electrostatic potentials. Blue surfaces are positively-charged and red surfaces negatively-charged. (**C**) Enlarged view focusing on TcsL 1–93 with labels pointing the amino-acids subjected to mutagenesis.

TcsL-cat consists of a compact core of α-helices (amino acids 94–543) containing the catalytic site DXD and an extended *N*-terminal bundle of four helices (amino acids 1–93). Hydrophobicity analysis revealed positively-charged residues on the tip of the four-helix bundle surrounded by hydrophobic amino acids which extend along the helices (Figure 1). The hydrophobic patches on one surface of the four-helix bundle with positively charged residues on the top represent a candidate binding sites for the acyl chains and polar head group, respectively, of negatively charged phospholipids. The following positions encompassing positively charged residues (blue) on the tip of helices and adjacent hydrophobic amino acids (yellow or orange) were selected to raise mutants Q10, K11, Y14, V15, K16, F17, R18, Q20, S38, R68, and Y78 (Figure 1).

### 2.2. Interaction of TcsL-cat Mutants with Brain PS

**Figure 2:**
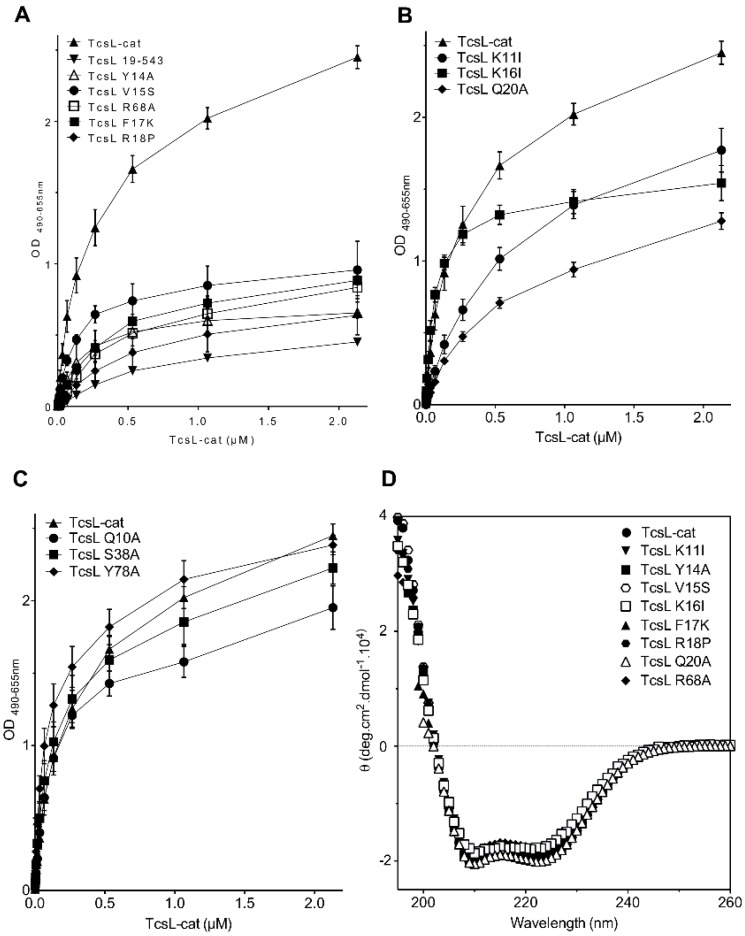
Interaction of the TcsL-cat mutants with BPS as monitored by ELISA. (**A**) TcsL-cat mutants with a drastic decrease in binding to BPS as compared to the deletion of the 18 *N*-terminal residues (TcsL 19–543); (**B**) TcsL-cat mutants with a moderate decrease in binding to BPS; (**C**) TcsL-cat mutants without significant change in binding to BPS; (**D**) FAR UV spectra of TcsL-cat mutants and TcsL wild-type. Spectra of TcsL-cat mutants with a drastic (Figure 2A) and moderate (Figure 2B) decrease in binding to BPS are shown. Proteins were diluted in Hepes 20 mM, NaCl 150 mM, TCEP 1 mM, pH 7.4.

TcsL-cat was found to bind with high affinity to membranes containing brain PS (BPS) as well as to BPS coated on plastic plates and monitored by ELISA [37]. Thereby, the TcsL-cat mutants were tested for their binding activity to BPS by ELISA (Figure 2). Deletion of the 18 *N*-terminal residues (TcsL-19–543) drastically reduced the TcsL-cat binding activity to BPS as previously shown [38]. Amino acid changes at positions Y14, V15, F17, R18, and R68 also strongly decreased the binding to BPS, albeit not to an extreme extent compared to TcsL-19–543 (Figure 2A). Mutations at positions K11, K16, and Q20 induced a moderate decrease in the interaction with BPS, whereas changes in Q10, S38, and Y78 did not significantly influence the binding to the phospholipid (Figure 2B,C).

The TcsL-cat mutants were checked for possible conformational change by circular dichroism spectroscopy. As shown in Figure 2D, all the mutants retained the same FAR UV spectrum profile compared to TcsL-cat wild-type, indicating that the amino acid changes did not significantly modify the protein conformation.

### 2.3. Localization of the PS Binding Site on TcsL

**Figure 3:**
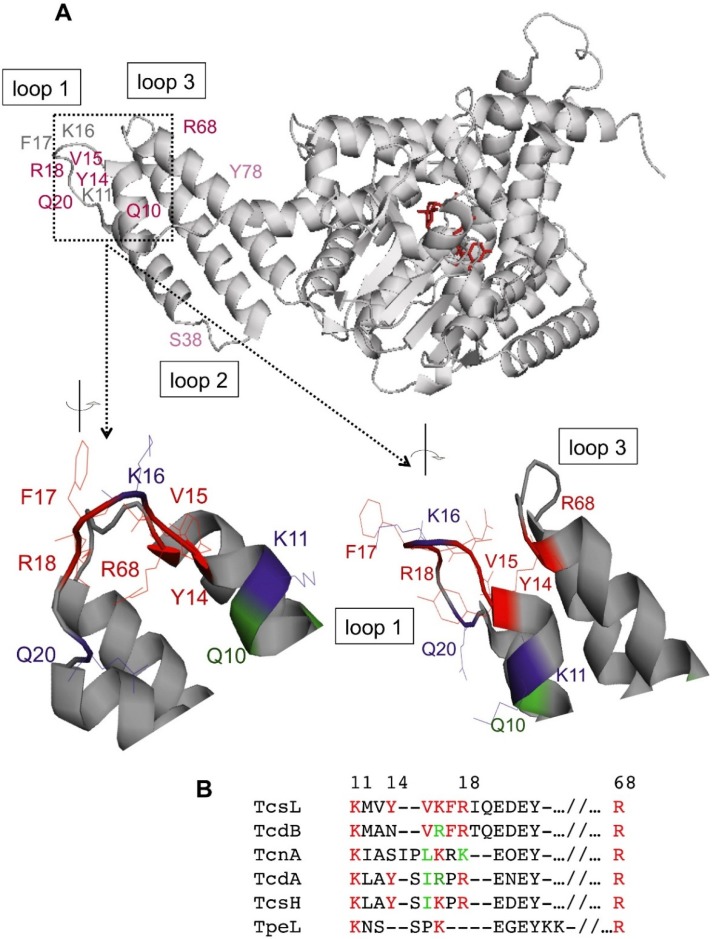
Localization of the critical residues involved in the interaction with PS. (**A**) The interaction site is located on the 10–20 amino acid pocket. Amino acids that are essential for the interaction with PS are shown in red and the amino acids that are implicated in the interaction in purple. Amino acids that are not implicated in the interaction are shown in green; (**B**) alignment of the amino acids critical for TcsL-cat binding to PS in the other toxins of the LCGT family. Amino acids in red are those which have been found critical for TcsL-cat binding to PS and which are conserved in the other toxins, and in green conservative amino acids. Amino acid numbering is that of TcsL.

Amino acids (Y14, V15, F17, R18, and R68) whose changes induced the most dramatic decrease in binding to BPS, are localized on the tip of the *N*-terminal four helix bundle (Figure 3). Amino acids (K11 and Q20) with a moderate effect on BPS binding flank the exposed residues on the helix tip. However, the positively-charged K16 albeit on the tip of the helix bundle (Figure 3A), had only a moderate interaction with BPS (Figure 2B). The amino acids (Q10, S38, and Y78) showing no significant alteration of BPS binding when changed with Ala (Figure 2C), are distant from the helix tip. Taken together, these results support that the tip of the *N*-terminal four-helix bundle in TcsL is critical for the interaction with BPS (Figure 3A). This structure is conserved in all LCGTs (discussed in [37]). The amino acids K11 and R68, which are critical for the BPS binding, are conserved in the other toxins of the LCGT family (Figure 3B). The residues V15 to R18 are conserved or have conservative substitutions in the other toxins, except in TpeL, which shows a different arrangement of the positively-charged residues in this region (Figure 3B). Regarding the amino acids involved in BPS binding of TcsL-cat, TcdB is the most related to TcsL, whereas TcnA, TcdA, and TcsH show more amino acid variations, notably F17 of TcsL is conserved in TcdB and is replaced by a Pro in the other three toxins (Figure 3B). These amino acid differences might impact the variable binding affinity to PS or PA according to the toxins as already observed [37].

### 2.4. Binding Site to BPS Is Required for TcsL-cat Localization to Cell Membrane

**Figure 4:**
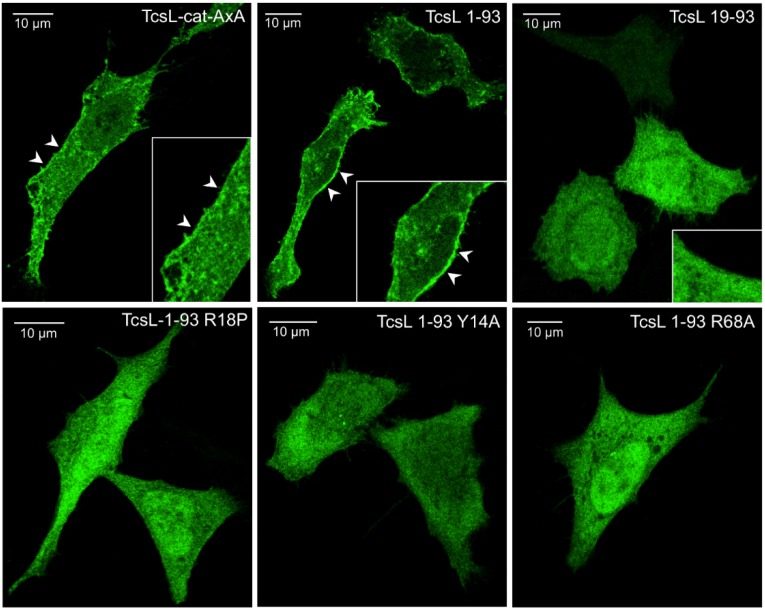
Localization of TcsL-cat in HeLa cells. HeLa cells were transfected with TcsL-cat-AxA, TcsL 1–93 or mutants in fusion with GFP and imaged in confocal microscopy 24 h after transfection. The inset shows a magnification of cell membranes. Bars represent 10 µm. The enzymatically inactive TcsL-cat-AXA as well as the 1–93 domain localized at the plasma membrane, whereas the truncated 19–93 domain and the 1–93 mutants R18P, Y14A, and R68A showed no specific binding to the plasma membrane.

The *N*-terminal four-helix bundle, also called the membrane localization domain (MLD), has been found to interact with the membrane [33]. We investigated the membrane localization of TcsL 1–93 and mutants in HeLa cells upon transfection of GFP-(green fluorescent protein)-fusion constructs and confocal microscopy. As a control, the whole enzymatically-inactive TcsL-cat with AXA mutation, instead of the enzymatic site DXD, was produced in fusion with GFP and transfected into HeLa cells. TcsL-cat-AXA localized at the inner face of the plasma membrane as visualized in confocal microscopy (Figure 4). TcsL 1–93 also showed the same pattern of localization to the membrane. In contrast, the TcsL-19–93 and TcsL 1–93 mutants abolished in binding to BPS (R18P, Y14A, and R68A) were not visualized on cell membranes (Figure 4). This confirms that the TcsL 1–93 domain is involved in the localization of the catalytic domain to the cell membrane and indicates that the BPS binding site is critical for the interaction with membrane phospholipids.

Binding affinity to BPS of wild-type TcsL-cat, TcsL-cat (AXA), and TcsL 1–93 was investigated by ELISA (Figure S1). Mutations on the enzymatic site (AXA) did not modify the affinity of the whole catalytic domain to BPS. The 1–93 domain showed a slightly reduced binding affinity to BPS. However, the 1–93 domain was able to localize to cell membrane (Figure 4).

### 2.5. Effects of TcsL-cat Mutants on Cell Viability

**Figure 5:**
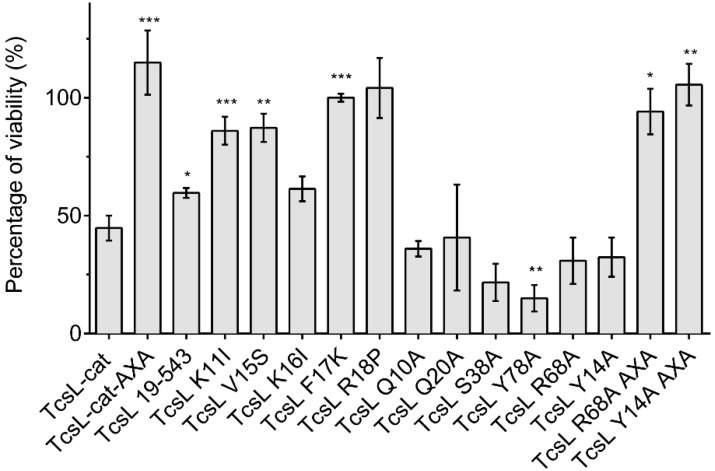
Effect of TcsL-cat or TcsL mutants treatment on cell viability. Cells were electroporated with TcsL-cat or mutants and proliferation tests were performed 72 h after electroporation. Data are expressed as mean ± SEM of at least three independent experiments. Statistically significant differences between results were evaluated by t test with Welch correction. A *p* value of <0.05 was considered statistically significant (* *p <* 0.05, ** *p <* 0.005, *** *p <* 0.0005).

The effects of TcsL-cat mutants on cell viability were monitored by electroporation of the purified proteins in HeLa cells and proliferation assay with methylthiazolyldiphenyl-tetrazolium (MTT) test (Figure 5). Wild-type TcsL-cat induced a marked viability reduction, whereas the enzymatic AXA mutant did not. The truncated TcsL-19–543 was moderately reduced in cytotoxic activity, but the mutations on K11, V15, F17, R18, and to a lower extent K16 markedly impaired TcsL-cat deleterious effect on cell viability. In contrast, amino acid changes at positions Y14, Q10, Q20, S38, R68, and Y78 did not modify or slightly increased TcsL-cat cytotoxicity (Figure 5). Among this latter category, the influence of mutations at positions Y14 and R68 was further investigated by analysis of the effect of an additional mutation (AXA) which inactivates the enzymatic site. It is noteworthy that double mutants TcsL Y14A AXA and TcsL R68A AXA were no longer cytotoxic. These findings support the notion that the cytotoxic activity of these TcsL-cat mutants was only due to UDP-glucosylation of Ras/Rac-GTPases.

### 2.6. Glucosylation Activity of TcsL-cat Mutants

**Figure 6:**
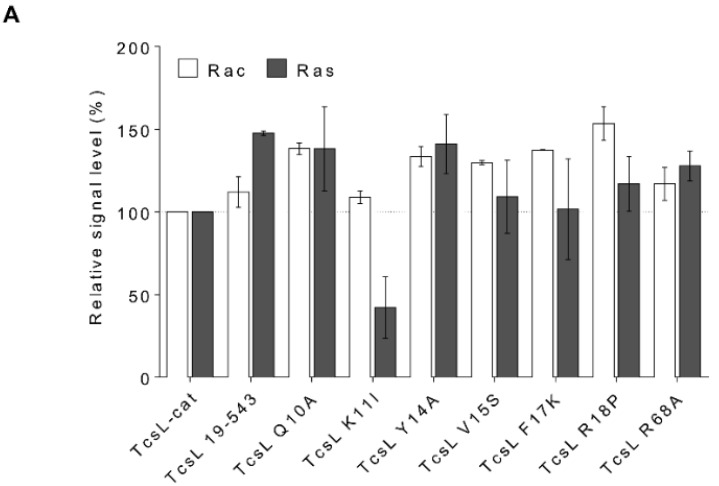

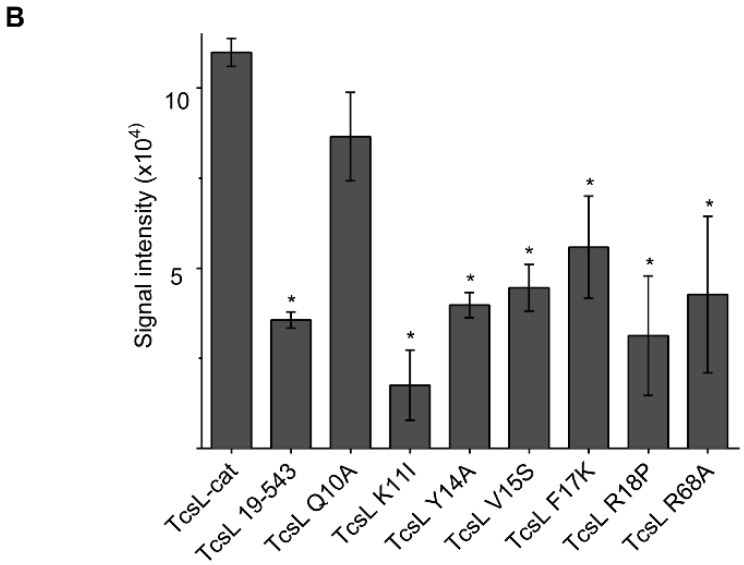
*In vitro* glucosylation activity of TcsL-cat and mutant towards Rac and Ras. (**A**). TcsL-cat and mutant glucosylation of Rac or Ras in solution. Rac or Ras proteins (1 μg) were incubated with 10^−7^ M of TcsL-cat or mutants for 15 min at 37 °C in the presence of UDP-[_14_C]glucose, and then run on SDS-PAGE. Then the gel was dried and autoradiographed. The bands were analyzed using an ImageJ gel analyzer. Data represents the mean ± SEM of at least three independent experiments. (**B**). TcsL-cat and mutants’ glucosylation of membrane-anchored Ras. Membrane-anchored Ras (7 ng) were incubated with 10^−7^ M of TcsL-cat or mutants for 15 min at 37 °C in the presence of UDP-[_14_C]glucose and then run on SDS-PAGE. Then the gel was dried and autoradiographed. The bands were analyzed using an ImageJ gel analyzer. Data represents the mean ± SEM of at least three independent experiments. Statistically significant differences between results were evaluated by one-way Anova. A *p* value of <0.01 was considered statistically significant (* *p <* 0.01).

*In vitro* glucosylation activity of TcsL-cat mutants was checked with purified soluble Ras and Rac proteins. The truncated TcsL-cat (19–543) and all the single amino acid mutants retained the same level of glucosyltransferase activity than the wild-type TcsL-cat (Figure 6A). However, whereas TcsL-cat-K11I showed a same enzymatic activity towards Rac than the TcsL-cat wild-type, this mutant weakly modified Ras (Figure 6A).

**Figure 7:**
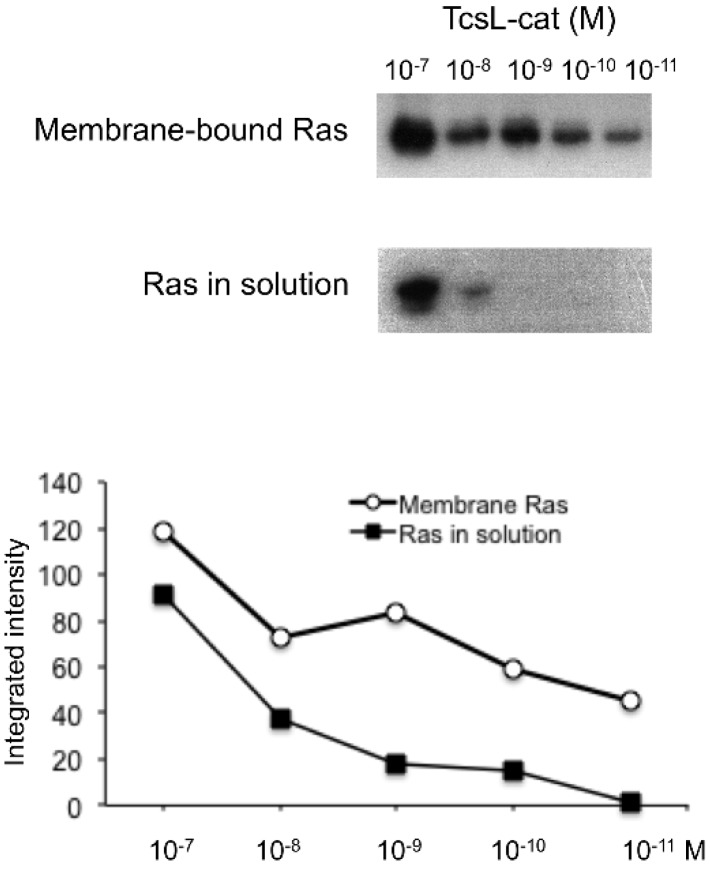
Glucosylation activity of TcsL-cat wt towards Ras in solution *versus* cellular membrane extracts enriched in Ras. Recombinant Ras (7 ng) or membrane-bound Ras (7 ng as assayed by ELISA) were used in each glucosylation test with serial dilutions of TcsL-cat in the presence of UDP-[_14_C]glucose. The reactions were performed at 37 °C for 30 min. One representative experiment from 3 is shown.

Binding of TcsL to membrane results in an increased glucosylation activity towards GTPase anchored to the lipid membrane. Indeed, binding of TcsL-cat to a PS-containing membrane increases by two orders of magnitude the rate of glucosylation of liposome-bound Rac compared to the enzymatic activity with substrate in solution [38]. We compared the enzymatic activity of TcsL-cat with Ras in solution *versus* membrane-bound Ras using cell membrane extracts enriched in Ras (Figure 7). The same amount of Ras (7 ng) as assayed by ELISA and 10-fold serial dilutions of TcsL-cat was used in each glucosylation test. An increase of three orders of magnitude of TcsL-cat enzymatic activity was observed with membrane-bound Ras compared to soluble recombinant Ras (Figure 7).

Then, we checked the glucosylation activity of TcsL-cat mutants with cell membrane-bound Ras. As illustrated in Figure 6B, the truncated TcsL-cat-19–543 and the TcsL-cat mutants (Y14A, V15S, F17K, R18P, and R68A) which showed a decreased binding to BPS (Figure 2A), and also induced a lower glucosylation rate towards membrane-bound Ras (Figure 6B). The mutant TcsL-cat K11I which had a lower activity with Ras in solution (Figure 6A) and a weakly modified membrane-bound Ras (Figure 6B). In contrast, the TcsL-cat mutants, which bound to BPS (Figure 2C), retained a similar level of glucosylation towards membrane-bound Ras than TcsL-cat (by weight) (Figure 6B and data not shown).

### 2.7. Model of TcsL-Cat Interaction with PS

**Figure 8:**
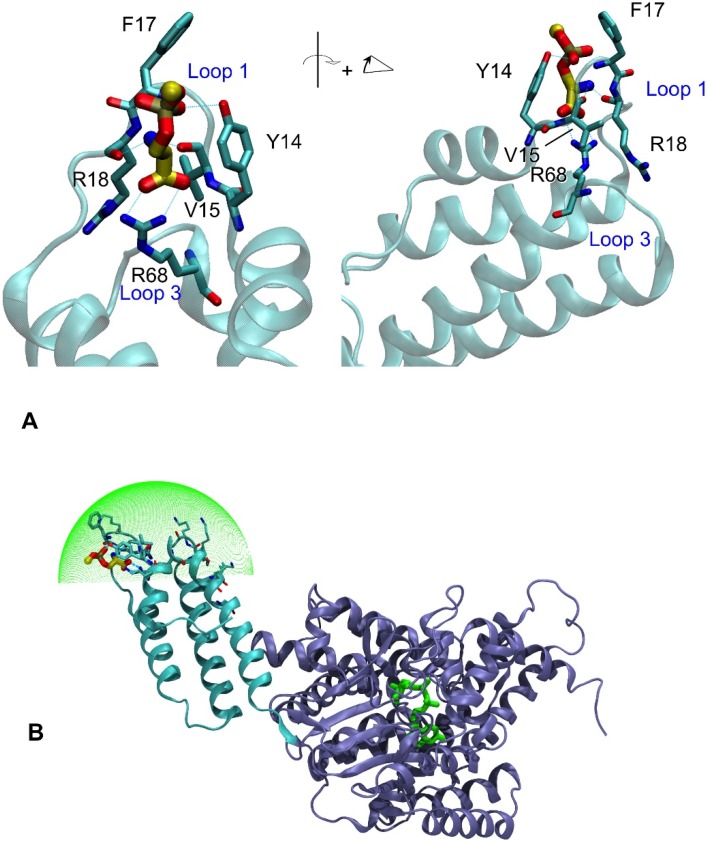
Structure modeling of TcsL-cat interaction with PS. (**A**). Structure modeling of the interaction of the *N*-terminal helix bundle tip with PS. Residues of loops 1 and 3 interacting with serine of PS are shown, dark blue, nitrogen; red, oxygen; cyan/yellow, carbon. The sphere (yellow) on the top of serine is the 1st carbon of glycerol linked to phosphate. Modeling was performed with FlexX within LeadIt with default settings [41]. PS was modeled with different lipid length, from DLPS down to a methyl-phospho-serine as depicted here with similar results for the positioning of the seryl group. (**B**). Modeling of TcsL-cat interaction with PS containing membrane. Lysine amine 11, 16, 64, 65, 70, 73, 74, and 76 and the methyl of the methyl-phospho-serine lied within 1.5 Å of a hemi-sphere identified by minimizing the χ^2^ for the distance of the atoms to the sphere. The green sphere represented has the same center, and a radius increased by 2 Å for clarity.

Mutations had been introduced in three identified small pockets of the 1-93 sub-domain. Mutations S38A and Y78A, each flanking a different pocket, showed no effect, thus implying PS should be binding in the third one (mutations Y14A, V15S, F17K, R18P, R68A, and flanking Q10A, K11I, Q20A). *In silico* modeling has been performed to dock PS on TcsL-cat. PS docked at the tip of the four-helix bundle, in the loop 1 between the helices 1 and 2, forming the third cavity (Figure 8A). Interestingly, L-serine fit well the binding site, but D-serine much less. The structure modeling shows a pocket formed by Y14, V15, and R18 with R68 from loop 3 at the bottom and Q10, A13 and Q20 flanking it (Figure 8A). The carboxyl group of PS would make a pair of hydrogen bonds (HB) with the guanidinium of R68 inserted in a notable position in the core of the sub-domain. Then, the PS amine could make HBs with the carbonyls of V15 and R18, in agreement with the strong impacts of V15A mutation, likely to impact the tight positioning of that amino acid and that of R18P, likely to impact the main chain conformation, and at least the carbonyl placement. Mutations Y14A, and F17K could impact the ability of the module to insert aromatic anchors to approach the PS head. Noticeably, the tip of the phosphatydyl (phosphoester glycerol carbon) and amines of lysines 11, 16, 64, 65, 70, 73, 74, and 76, lie at the tip of subdomain 1–93 with an approximate hemi-spherical distribution. This positioning supports that TcsL-cat would encounters the negative PS-lipid membrane through the binding of the tip of the *N*-terminal four-helix bundle to the polar head group of PS (Figure 8B), but would be facilitated by some local fluidity of the membrane to accommodate the geometry of the charge distribution of the encounter tip. Once the first contact established, the global distribution of charges and hydrophobic residues may lead to further reorganization of the helices at the membrane surface.

## 3. Discussion

We have previously found that TcsL-cat binds preferentially to PS and that the TcsL enzymatic activity is at least 100-fold higher when Rac is anchored to a lipid membrane containing PS than when Rac is in solution [38]. Furthermore, TcsL-cat glucosylation activity was increased by three orders of magnitude on membrane attached Ras as compared to Ras in solution (Figure 7). Binding of the toxin enzymatic domains to the membrane increases the local concentration of toxin in a close proximity to the substrates and possibly in an optimal orientation, thus facilitating a high enzyme activity level. The 1–93 *N*-terminal domain extends as a separate four helix bundle from the core of LCGT catalytic domain which consists in a compact structure of α-helices and β-strands containing the catalytic site DXD (rev in [20]), and is involved in membrane insertion. However, while the LCGT 1–93 domain drives the binding to a wide range of phospholipids [33,38], the whole catalytic domain retains a specific interaction with PS and phosphatidic acid. Moreover, the binding affinity of TcsL 1–93 domain to brain PS is lower (about 20-fold less) than that of TcsL-cat [37], indicating that the catalytic core domain also modulates the interaction with the phospholipids.

### 3.1. Identification of the PS Binding Site on the Tip of the N-Terminal Four-Helix Bundle

To better define the interaction of TcsL-cat with PS, we have generated mutations in the 1–93 domain. The analysis of the 1–93 domain structure indicated the presence of positively-charged residues (blue) on the tip of the helix bundle and on one face, while the negatively charged amino acids (red) are located on the opposite surface (Figure 1 and [37]). Hydrophobic patches (yellow or orange) flanked by positively charged residues lie along helices 3 and 4 (Figure 1). Mutations were performed on amino acids mainly located on the tip and flanking regions of the *N*-terminal four-helix bundle, in particular trying to identify a serine binding site that could accommodate the head of PS (Figure 3). As already previously found [38], the deletion of the 18 *N*-terminal residues prevented the TcsL-cat binding to BPS as monitored by ELISA (Figure 2). Residues A13 to E21 constitute the loop 1 which is exposed at the tip of the four-helix bundle between helices 1 and 2 [39]. Substitution of the positively-charged residues (K16 and R18) at the tip of loop 1 had different effects. K16 induced a moderate decrease in BPS binding, possibly because of an already strong insertion of Y15 and F17. Conversely, R18 induced a strong decrease in BPS binding plausibly by its impact on the main chain positioning. Accordingly, mutation of the aromatic amino acids F17 and Y14, as well as the aliphatic V15 in central positions of loop 1, which also drastically impaired the binding to BPS. This may be due to the packing of the valine side chain in the tip of the bundle, reinforcing its positioning in an allowed region of the Ramachadran plot (−124, 119) of this residue. Alongside, substitution of the terminal loop 1 residue (Q20) at the connection with helix 2 moderately altered the binding. Amino acids in the central part of the loop 1 are likely the most exposed to the substrate. In contrast, amino acid changes in the helix 1 (Q10, K11) or helix 4 (Y78) did not significantly influence the binding affinity to BPS. Amino acid change of the positively-charged residue (R68) in the loop 3 between the helices 3 and 4 which is closely located to loop 1 on the tip of the four-helix bundle prevented the binding to BPS, whereas substitution of S38 in the opposite loop 3 (Figure 1 and Figure 3) did not modify the binding, hence pointing towards the loop-1 loop-3 for the membrane first contact.

Thereby, the tip of the *N*-terminal four-helix bundle constituted of loops 1 and 3 which are rich in positively-charged residues, plays a major role in the binding to BPS. Nonetheless, this non-planar distribution of potential first encounter anchors is in good agreement with the finding that TcsL-cat does not bind well on rigid PS membranes (e.g., DPPS) [37]. Structural modeling shows that Y14, V15, F17, and R18 of loop 1 and R68 of loop 3 form a pocket which accommodates the binding to L-serine (Figure 8) in agreement with the alteration of PS binding when these residues were mutated. This supports the assertion that TcsL-cat binds to membranes via interaction of the tip of the *N*-terminal four-helix bundle with the polar head group of PS and that the PS binding site is mainly localized in the loop 1 (Figure 8). Moreover, the acyl chains of phospholipids have also been found to influence the interaction of TcsL-cat with membranes [37]. The hydrophobic face of the *N*-terminal domain (Figure 1) would represent an appropriate candidate binding site for the acyl chains. However, it is unlikely that TcsL-cat inserts deeply in the membrane and interacts directly with the acyl chains. Binding assays of various acyl chain derivatives of phospholipids suggest that acyl chains modulate TcsL-cat binding mainly by influencing the membrane fluidity [37], in agreement with the positioning requirement of the PS binding site on the tip side of the 1–93 subdomain. In addition, it has been found that the *N*-terminal 1–93 domain interacts with a wide range of phospholipids but with a lower affinity than the whole TcsL-cat which specifically binds to PS containing membrane [37]. Therefore, we propose that TcsL-cat binds to the polar head group of PS in phospholipid membrane via the tip of the *N*-terminal four-helix bundle and that the structure of the helix bundle is reorganized on the surface of the lipid bilayer. Short and/or unsaturated phospholipid acyl chains, which enhance membrane fluidity, facilitate a partial insertion of the four-helix bundle presenting lysine amines to the negatively-charge head groups of the membrane before the establishment of hydrophobic interactions. Specific binding of the whole TcsL-cat to PS containing membrane requires a stronger interaction than the hydrophobic effect between the four-helix bundle and phospholipids. The specific recognition of the PS polar head group by the PS binding site in TcsL-cat *N*-terminus appears to assist membrane binding by building up the necessary affinity. It is not clear whether additional residues in the enzymatic core are required for the specific binding to PS as suggested by the specific interaction of the whole TcsL-cat *versus* the 1–93 domain [37], but this could be mediated by the patch of hydrophobic amino-acids at the interface between the 1–93 and the rest of the cat domain, for example.

### 3.2. The TcsL-cat N-Terminal Domain Is Involved in Membrane Location and Modulates the Enzymatic Activity towards the Membrane-Bound Substrates

The main function of the *N*-terminal four-helix bundle was assigned to the sublocalization of the catalytic domain to the inner face of the plasma membrane [33]. Here, we observed that the TcsL 1–93 domain delivered into the cell by electroporation localized to the plasma membrane. Conversely, mutants impaired in BPS interaction also lost their membrane binding (Figure 4). This further substantiates the ability of the TcsL *N*-terminal domain to bind to the membrane and that the membrane binding is supported by the recognition of PS. To analyze the role of the binding to PS in the mode of action of TcsL-cat, we monitored the enzymatic activity and cytotoxicity of wt and TcsL-cat mutants. All of the mutants, except K11I (see below), retain the same glucosylation level of Rac or Ras in solution as that of TcsL-cat. Therefore, binding to PS does not directly interfere with the substrate recognition and the intrinsic glucosyltransferase activity of TcsL-cat. However, in contrast to TcsL-cat, all the mutants impaired in the recognition of PS showed a weak enzymatic activity towards membrane-bound Ras (Figure 6B). A comparable glucosylation level was observed with Ras in solution *versus* membrane-bound Ras for these mutants, further demonstrating that the increased activity of TcsL-cat with membrane-bound substrate is not due to an enhanced intrinsic enzyme activity but to TcsL-cat binding to the membrane via PS with subsequently high local enzyme concentration.

Interestingly, the K11I mutant retained the specific interaction with Rac, but showed a marked decrease in the recognition of Ras. The LCGT substrate binding site has been determined in the *C*-terminal part of LCGT catalytic domain. Indeed, the TcsL residues 364–408 have been found to mediate the recognition of Rac/Cdc42, and the residues 408–516 that of Ras [42,43]. Our results indicate that the specific TcsL-cat interaction with Ras also depends on *N*-terminal amino acids such as K11.

Another outcome from the K11I mutant results including a decreased activity towards Ras, a conserved activity towards Rac, and a lack of cytotoxicity despite the fact that it still bound to PS, is that it confirms the pivotal role of the Ras pathway in the TcsL-induced cytotoxicity.

### 3.3. TcsL-cat Binding to Membrane in Appropriate Orientation Is Essential for Efficient Modification of Membrane Bound Ras and Subsequent Cytotoxicity

In addition to actin cytoskeleton alteration, TcsL causes cell death by apoptosis. TcsL-induced Ras inactivation by glucosylation and subsequent inhibition of the PI3K/AKT signaling pathway is considered as the TcsL-dependent mechanism of cytotoxicity [21]. TcsL-cat mutants V15S, F17K, and R18P were not cytotoxic upon transfection by electroporation into cells supporting that binding to the membrane via PS with subsequently high levels of membrane-bound Ras glucosylation is essential for TcsL-cat cytotoxicity. However, the mutants Y14A and R68A were cytotoxic, albeit they were impaired in the interaction with PS and did not induce a high glucosylation level towards membrane-bound Ras *in vitro*. It was checked that the cytotoxic activity of these mutants were dependent of their glucosylation activity since the same mutants with inactive glucosylation site were no longer cytotoxic (Figure 5). Albeit, the 1–93 domains of Y14A and R68A were not visualized to cell membranes (Figure 4), it cannot been be ruled out that TcsL-cat Y14A and R68A interacted sufficiently with the plasma membrane to induce an appropriate Ras inactivation level leading to cytotoxicity. Indeed, the amino acids V15, F17, and R18, whose mutations resulted in inhibition of cytotoxicity, are localized in the central part of loop 1 forming a cavity for serine binding, whereas Y14 and R68 lie at one extremity of loops 1 and 3, respectively. This suggests that the residues in the central part of loops 1 and 2 play a major role in TcsL-cat interaction with phospholipids, whereas those at the loop extremities might represent lower affinity binding sites. Substitutions of the loop boundary residues might not completely prevent the interaction of the loop central part with PS in the *in vivo* conditions. The effects of the mutation on cell viability may also involve subtle impact on the TcsL-cat orientation that is still to be explored.

## 4. Conclusions

Taken together, these results show that the loops 1 and 3 on the tip of the *N*-terminal four-helix bundle of TcsL, which are rich in positively-charged residues, constitute a critical binding site for PS, and that binding to membranes via PS is required to direct the full enzymatic activity of TcsL-cat towards the membrane-bound substrates Ras and Rac triggering cell death. The tip of the four-helix bundle is probably involved in the interaction with the head group of negatively-charged phospholipids, whereas the first methyl groups of the acyl chains and the hydrophobic face of the TcsL helix bundle interact via hydrophobic forces. The four-helix bundle could also have a role to play in the orientation of the whole TcsL-cat domain so that it can interact with its substrates, Ras and Rac. It is not excluded that binding sites also lie in the enzymatic core ensuring the high specificity of TcsL-cat for PS and PA. Since the structure of the catalytic domains including the *N*-terminal four-helix bundles are conserved in LCGTs, all the toxins of this family likely share a common mechanism of membrane binding required for their full toxic activity. However, while most of the amino acids which are essential for membrane binding in TcsL are conserved or have conservative substitutions in the other toxins of the LCGT family, some differences can be observed (Figure 3B), notably in TpeL which might account for different binding affinity towards PS or PA according to the toxin as already reported [37].

## 5. Experimental Procedures

### 5.1. Cloning and Protein Production

The catalytic domains corresponding to amino acids 1–543 (TcsL 1–543), 1–93 and 19–543 from TcsL-82, were cloned in a pET28 vector (Merck-Novagen, Saint-Quentin-en-Yvelines, France) as described previously [38]. Site-directed mutagenesis was performed by PCR or inverse PCR using pCR2.1-based constructs as a template and appropriate primers. The mutated genes were then transferred into pET28 vector. The constructs were expressed in *Escherichia coli* and purified by nickel chromatography followed by gel filtration (Figure S2). Proteins were stored at 4 °C in Hepes 20 mM, NaCl 150 mM, TCEP 1 mM, pH 7.4.

Recombinant Rac and Ras proteins were obtained as previously reported [8].

### 5.2. ELISA Test with Immobilized Phospholipids

Wells of microtiter plates (Nunc maxisorp; Nunc, Roskilde, Denmark) were coated with 12.1 nmol brain phosphatidylserine (BPS, 840032C, Avanti Polar Lipids (Avanti Polars Lipid Alabaster, AL, USA)) diluted in ethanol by evaporation at room temperature. After blocking with 30 mg/mL BSA in carbonate buffer (0.015 M Na_2_CO_3_, 0.035 M NaHCO_3_ pH 9.6), the wells were incubated with various amounts of TcsL (wild-type or mutants) in PBS tween 20 0,1% BSA 1% for 1 h at room temperature. Thereafter, the wells were incubated with rabbit anti-TcsL 1–546 serum (1:1000) in PBS tween 20 0.1% BSA 1% for 1 h at room temperature then with protein A-peroxidase (1:3000, Biorad, Marnes-la-Coquette, France) for 1 h at room temperature. For revelation, 100 µL of 1 mg/mL ortho-phenylene-diamine in citrate buffer (0.05 M Na_2_HPO_4_, 0.02M citrate) is used. Color development was stopped after 12 min by adding 50 µL HCl 3 M and absorbances at 490 nm and 655 nm were measured with a microplate reader (Biorad, model 680). To account for nonspecific binding, binding of TcsL to uncoated wells was determined in each experiment and subtracted from the binding to immobilized phospholipids. *K*d and *B*max were calculated using GraphPad (Prism GraphPad Prism, Ritme, Paris, France).

### 5.3. Circular Dichroism (Far-UV) Spectroscopy

CD spectra were recorded on an Aviv 215 spectropolarimeter (Aviv Biomedical, Lakewood, NJ, USA) with protein samples at 0.7–1.4 mg·mL^−1^ in Hepes 20 mM NaCl 150 mM TCEP 1 mM. Far-UV CD spectra were recorded between 195 and 260 nm using a cylindrical cell with a 0.02 cm path length, an averaging time of 1 s per step and bandwidth of 1 nm. Three consecutive scans from each sample were merged to produce an averaged spectrum and corrected using buffer base lines measured under the same conditions. Data were normalized to the molar peptide bond concentration and path length and expressed as mean residue ellipticity ([θ] degree·cm^2^·dmol^−1^) as described elsewhere [44].

### 5.4. Construction of TcsL AXA, TcsL 1-93, TcsL 19-93, and TcsL 1-93 Mutants with GFP-Fusion

The catalytic domains corresponding to amino acids 1–543 (TcsL 1–543), 1–93 and 19–93 from TcsL-82, were cloned in a pEGFP-C1 vector (Clontech, Saint-Germain-en-Laye, France) in BglII and PstI restriction sites. Site directed mutagenesis was performed by PCR or inverse PCR using pCR2.1- or pUC19-based constructs as a template and appropriate primers. The mutated genes were then transferred into the pEGFP-C1 vector.

### 5.5. Cell Line, Transfection, and Microscopy

HeLa cells (ATCC CCL-2) were cultured in Dulbecco’s modified Eagle medium (DMEM; Invitrogen, Courtaboeuf, France) supplemented with 10% foetal calf serum (FCS; Invitrogen) and 1% penicillin-streptomycin. HeLa cells were grown to approximately 70% confluence on coverslips in 12-well dishes then transiently transfected using X-tremeGene HP (Roche, Meylan, France) following the manufacturer’s protocol. 24 h after transfection, coverslips were washed with PBS and fixed in 4% formaldehyde in PBS 1X then mounted onto glass slides. Slides were imaged for epifluorescence with a confocal right Zeiss LSM700.

### 5.6. Delivery of TcsL-cat and Mutants into Cultured Cells by Electroporation

The recombinant TcsL-cat or TcsL mutant was delivered into HeLa cells by electroporation. HeLa cells were detached with versene and 10^6^ viable cells were incubated with 5 µg toxin in electroporation buffer (20 mM Hepes, 1 mM NaHPO4, 5 mM KCl, 140 mM NaCl, 10 mM glucose). The amount of protein used for electroporation was selected on the basis of dose-response proliferation assay results (data not shown). Cells were then incubated on ice for 10 min and electroporated at 160 V, 500 µF and infinite resistance (BioRad GenePulser X-cell), in 0.2 cm electroporation cuvettes (BioRad, Marnes-la-Coquette, France). Cells were placed on ice for 10 min then suspended in 2 mL of medium and 100 µL transferred in 96-well plate (TPP).

### 5.7. Proliferation Assay

Proliferation assays were performed with methylthiazolyldiphenyl-tetrazolium (MTT, Sigma Aldrich, L’Isle d’Abeau, France) solubilized in PBS 1X. We incubated 100 µL of cells with 50 µL of MTT solution for 3 h at 37 °C. Purple formazan crystals were then solubilized by incubation with 100 µL of solubilization solution at 37 °C overnight. Absorbance was measured at 570 nm.

### 5.8. In Vitro Glucosylation Assay

Glucosylation of small GTPases was achieved as followed. Two µL of UDP-[_14_C]glucose in ethanol (O.2 mCi, 300 mCi/mmol; DuPont NEN, les Ulis, France) was dried under vacuum; 1 µg of recombinant GTPase or 2 µg total protein of membranes of rat fibroblasts (Rat-1-EJ-Rap2.31.A8) overexpressing Ras [10] (7 ng) in a 20 µL final volume of glucosylation buffer (50 mM triethanolamine pH 7.5, 2 mM MgCl2, 0.3 mM GDP, 1 mM dithiothreitol, and 100 µg/mL bovine serum albumin) was incubated with 10^−7^ M recombinant TcsL-cat or mutants for 15 min at 37 °C. Ras amount was assayed by ELISA with Ras specific monoclonal antibody (Y13-259, Abcam, Paris, France). The reaction was stopped by adding 5 µL of 2X SDS sample buffer. The sample was then boiled and electrophoresed on a 15% SDS-polyacrylamide gel. After staining with Coomassie blue, followed by distaining, the gel was dried and autoradiographed.

## Supplementary Materials

The following are available online at www.mdpi.com/2072-6651/8/4/90/s1. Figure S1. Binding of TcsL-cat, TcsL-cat-AXA and TcsL 1–93 to BPS as monitored by ELISA analysis. (A) Interaction of the TcsL-cat and TcsL 1–93 with BPS. The values represent the means ± SEM of three independent experiments; (B) Affinity constants of TcsL-cat, TcsL-cat-AXA, and TcsL 1–93 binding to PS as determined by ELISA. The values represent the means ± SEM of three independent experiments, Figure S2. SDS-PAGE of recombinant TcsL-GT and mutants. Recombinant TcsL-cat and mutants (1–5 µg) have been run on a SDS-10%PAGE gel and stained with Coomassie blue.
