# Supplementary material for: The Tip of the Four N-Terminal α-Helices of Clostridium sordellii Lethal Toxin Contains the Interaction Site with Membrane Phosphatidylserine Facilitating Small GTPases Glucosylation
Source: Toxins (Basel). 2016 Mar 25;8(4):90. doi: 10.3390/toxins8040090 (PMC4848617; doi:10.3390/toxins8040090)
Supplement: Supplementary file 1 [file toxins-08-00090-s001.pdf]

Supplementary Materials: The Tip of the Four *N*-Terminal α-Helices of *Clostridium sordellii* Lethal Toxin Contains the Interaction Site with Membrane Phosphatidylserine Facilitating Small GTPases Glucosylation

Carolina Varela Chavez, Georges Michel Haustant, Bruno Baron, Patrick England, Alexandre Chenal, Serge Pauillac, Arnaud Blondel, Michel-Robert Popoff

A

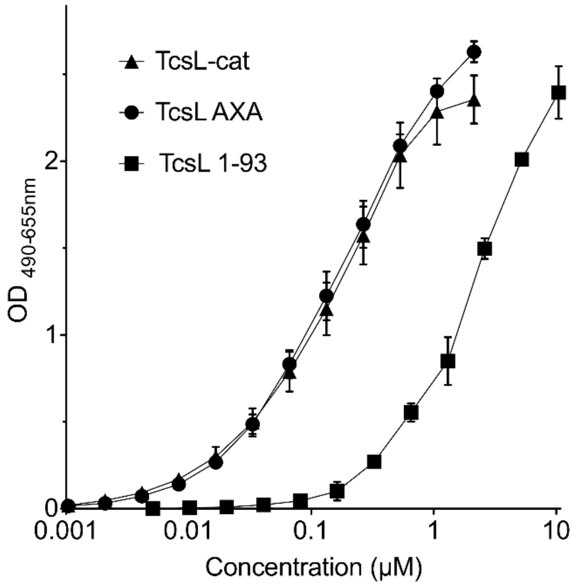

B

	TcsL-cat	TcsL AxA	TcsL 1-93
Bmax (uOD)	3.4 ± 0.5	3.5 ± 0.6	3.3 ± 0.4
Kd (μM)	0.9 ± 0.2	0.9 ± 0.5	3.5 ± 0.8

Figure S1. Binding of TcsL-cat, TcsL-cat-AXA and TcsL 1–93 to BPS as monitored by ELISA analysis. (**A**) Interaction of the TcsL-cat and TcsL 1–93 with BPS. The values represent the means ± SEM of three independent experiments; (**B**) Affinity constants of TcsL-cat, TcsL-cat-AXA, and TcsL 1–93 binding to PS as determined by ELISA. The values represent the means ± SEM of three independent experiments.

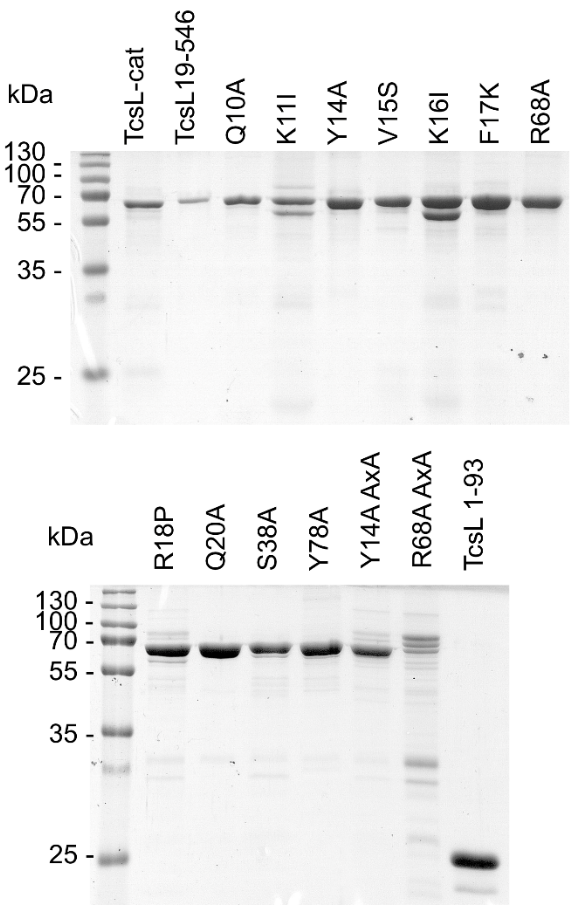

Figure S2. SDS-PAGE of recombinant TcsL-cat and mutants. Recombinant TcsL-cat and mutants (1–5 µg) have been run on a SDS-10%PAGE gel and stained with Coomassie blue.
